# Determinants of awareness and implementation of five-stage lesson plan framework among Christian Religious Education teachers in Meru County, Kenya

**DOI:** 10.1016/j.heliyon.2022.e11177

**Published:** 2022-10-26

**Authors:** Victor Okoth Saoke, Zachary N. Ndwiga, Pauline W. Githaiga, Collins M. Musafiri

**Affiliations:** a Department of Education, University of Embu, PO BOX 6-60100, Embu, Kenya; b Department of Curriculum, Instruction and Educational Management, Egerton University, PO BOX 536, Egerton-Njoro, Kenya; c Cortile Scientific Limited, PO Box 34991-00100, Nairobi, Kenya

**Keywords:** Awareness, Implementation, Christian Religious Education, Five-stage lesson plan framework, Ordered probit model

## Abstract

Approaches, methods, and techniques of teaching Christian Religious Education (CRE) in Kenya have changed over time. Improved teaching strategies such as a five-stage lesson plan framework enhance students' performance. Despite the novelty of the framework, there is limited information on its spread and utilization among CRE teachers in Kenya. Therefore, this study assessed the determinants of awareness and implementation of the novel five-stage lesson plan framework in Meru County, Kenya. The study sampled 226 CRE teachers using a semi-structured questionnaire. The study employed an Ordered Probit Model to assess the determinants of the number of five-stage lesson plan stages awareness and implemented by the CRE teachers. The Ordered Probit analysis revealed that gender, academic qualification, working experience, and challenges were crucial determinants of awareness and implementation of the five-stage lesson plan framework. The findings implied that policymakers and relevant stakeholders in education should consider pre-service and in-service training, workshops, and seminars in upscaling or promoting the awareness and implementation of the five-stage lesson plan framework. Educational policies targeting improved teaching methods should enhance recognition and operation of the five-stage lesson plan framework.

## 1 Introduction

Effective teaching entails the success of an inspiring and motivating interactional process with the learners (Konst and Scheinin, 2018). It involves the implementation of different learning styles grounded on the learners' environment since they may have numerous learning experiences (Assefa and Mohammed, 2022). The teacher must be a fundamental ingredient contributing to teaching effectiveness (Makovec, 2018). Every teacher plays a significant role in the teaching and learning activities (Boiliu et al., 2021). In this pedagogical process, the mastery of content is insufficient. The teacher needs to be aware of the myriad factors that contribute to effective learning (Aimah and Purwanto, 2018). Awareness is the capability to perceive and recognize, sense and feel, or be mindful of objects and events (Gafoor, 2012). The awareness and implementation of the lesson’s sequencing are salient factors in enhancing content delivery (Kimosop, 2015). Therefore, teachers' awareness of innovative teaching practices could be essential in improving students' performance.

Human beings are growth-oriented, constantly eyeing different means of improving and attaining their potential (Vadivel et al., 2022). Consequently, innovative teaching approaches are essential in enhancing content delivery among teachers (Wambui and Amukowa, 2013). Innovation and technology are practical tools for all instructors and learners worldwide (Tilwani et al., 2022). Thus, employing innovative methodologies is critical in improving the quality of teaching (Santos et al., 2020). The shifting balance from conveying a recognized form of knowledge to preparing all-around learners has implications for pedagogy (Peterson et al., 2018). Due to cultural and economic diversities, learning desires, needs, and requirements are becoming increasingly diverse (Naz and Murad, 2017) The diversities among learners could be addressed by utilizing innovative and advanced teaching approaches during their classroom activities. Hence, teachers' innovativeness creates effective teaching and learning (Ikwuka and Henry, 2017). Therefore, innovation is a tool of positive change in curriculum implementation (Serdyukov, 2017). Furthermore, the curriculum implementation needs continuous innovation for sustainability.

**Figure 1:**
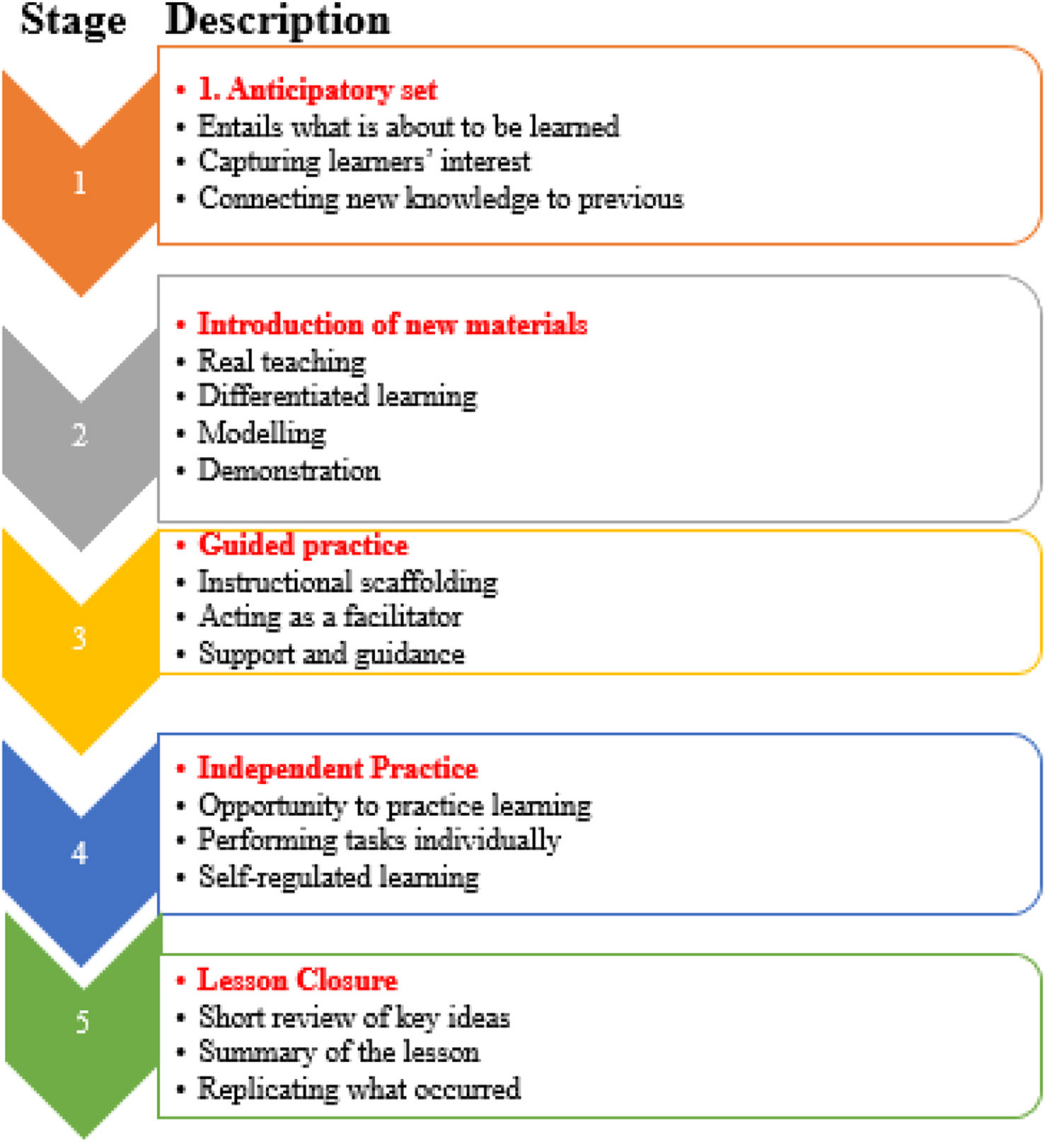
Five-stage lesson plan framework.

Approaches, methods, and techniques of teaching Christian Religious Education (CRE) in Kenya have changed over time (Buchanan, 2005). According to Kowino et al. (2011), teaching methods and strategies adopted by CRE teachers are probably answerable for the nature of teaching, which is similar to preaching. Therefore, CRE teaching has been connected to preaching for a long time. Improved teaching strategies such as a five-stage lesson plan framework enhance content delivery and students' performance (Figure 1). The framework is linked to the ideas of the Herbartian approach and Madeline Hunter lesson plan model, which are concerned with preparing learners to receive new knowledge (Deng, 2013).

The Five-stage Lesson Plan Framework has a long history, particularly the teaching through innovative pedagogy. It was predominantly established between the 1840s and 1990s through the ideas of Johann Friedrich Herbartian and Madeline Cheek Hunter (Friesen, 2020). The framework was first suggested by Herbart, a German Educator, in 1841. He developed a modern scientific pedagogy called the Herbatian Approach of Teaching. The approach recommended five recognized stages in content delivery: Preparation, presentation, association, generalization and application.

In 1994, Madeline Cheek Hunter developed a teaching model called Madeline Hunter Lesson Plan. Madeline’s lesson plan advocated for seven steps: aims, standards; anticipatory set; teaching; guided practice; closure, and independent practice (Deng, 2013). To avoid confusion, the followers of Herbartian and Madeline Hunter’s approaches worked together with the aim of learners' preparation to comprehend novel ideas. The recommended stages of the Herbartian and Madeline Hunter lesson plan were restructured by their followers to a conception of an instructive approach applicable to the different teaching spaces, the Five-Stage Lesson Plan Framework (Friesen, 2020). Thus, the FSLP framework was gradually established from Herbartian and Madeline Hunter’s methodologies. As the name suggests, stages are; anticipatory set, the introduction of new materials, guided practice, independent practice, and lesson closure (Figure 1).

The framework allows summarizing the instructional process and assessing comprehension. Other instrumental models include the Inquiry-based lesson plan, 5e model, predict-observe-explain model, and learning cycle model. These models are only effective in teaching subjects under the sciences cluster. They involve experimentation and hands-on activities (Namdar and Kucuk, 2018). However, the models are not applicable in teaching subjects under the humanities cluster. The FSLP framework is appropriate for teaching all the learning areas in the curriculum (Varzari and Ceh, 2015). Hence, the teaching and learning of CRE through the FSLP framework is not exceptional.

The FSLP framework assists learners in calling to mind the subject matter in both scientific and Humanities learning areas (Varzari and Ceh, 2015). Today, the significance of the FSLP framework in the teaching-learning process of all subjects cannot be disregarded. The exploration discoveries reinforced that the FSLP framework offers a tangible foundation for easy comprehension of scientific terms, ideas, and CRE concepts (Friesen, 2020). Accordingly, Kimosop (2015) asserts that teaching CRE requires appropriate planning by the teacher to achieve the expected learning outcomes anchored on emerging techniques. Similarly, using emerging approaches such as life skills and the FSLP strategies in teaching CRE remains unavoidable if formal learning is the target (Ngussa, 2015). These two models motivate learners to learn independently as the instructor is a facilitator. Kamaeva et al. (2022) assert that motivation and updated approaches alongside learning can move learners just as forces move objects.

Generally, there is no perfect technique for teaching and learning (Makovec, 2018). Nevertheless, numerous investigators currently approve that supplementary learner-centred teaching approaches such as the FSLP framework in the teaching space can advance learning and understanding. Applying a teacher-centred strategy leaves out several skills and understanding chances for learners (Serdyukov, 2017). The CRE instructors must be exhaustively equipped to apply this framework to implement the CRE curriculum. The framework will improve their capability to understand and implement the CRE curriculum aims by the Ministry of Education as specified in the Educational Program. Thus, implementing the FSLP framework in schools to teach all the learning areas is desirable.

Regardless of the changes in instructional practices, CRE still relies on old-style/traditional teaching approaches (Buchanan, 2005). However, the innovative content delivery methods are a considerable alarm in teaching CRE, but then again, the concept is narrow among the CRE instructors (Walaba and Kiboss, 2013). Therefore, CRE teachers are compared to preachers because they employ traditional methods of teaching, which are teacher-centred. These teaching methods are not suitable for the sequential delivery of the CRE content (Situma, 2016). Nevertheless, fresh and inventive teaching methods renew old-style teaching to advance the comprehension and utilization of new information delivered (Nurutdinova et al., 2016). Since the CRE teachers are not well exposed to the learner-centred approaches to content delivery, the study suggests the FSLP framework, an inventive pedagogical strategy founded on the different teaching styles to improve CRE instruction.

The framework is essential in improving content delivery and performance in CRE (Friesen, 2020). Instructors' characteristics and challenges influence the awareness and implementation of educational innovation (Serdyukov, 2017). Despite the importance of the FSLP framework on content delivery and enhanced performance, there is limited information on its awareness and adoption among CRE teachers in Kenya. Additionally, scientific studies on teachers' awareness and implementation of the framework in Kenya are scanty. Therefore, the study aimed to achieve the following objectives; to i) determine the awareness and implementation of the 5-stage lesson plan, and ii) assess the determinants of 5-stage lesson plan awareness and implementation among CRE teachers in Meru County, Kenya.

## 2 Methodology

### 2.1 Study area

We conducted the study in Meru County, Eastern Kenya. During the study, Meru County had 367 secondary schools across six sub-counties. The sub-counties are Imenti Central, Imenti South, Imenti North, Igembe East, Igembe West, and Buuri, which had 53, 59, 66, 63, 69, and 36 secondary schools, respectively. The study involved five sub-counties except for Buuri, where the piloting of the research instrument occurred. Therefore, this study involved 331 secondary schools, 307 public and 24 private. The schools were in the levels of national, extra county, county, and sub-county secondary schools such as 4, 21, 83, and 223. The county had 6520 secondary school teachers and 646 CRE teachers. The CRE teachers were spread as Imenti Central (102), Imenti South (125), Imenti North (140), Igembe East (131), and Igembe West (148).

### 2.2 Survey variables

The FSLP awareness and implementation were the dependent variables in this study. The variable was the number of the specific stages of the FSLP the teacher was aware of or implemented (Table 1). Therefore, awareness and implementation were 1 if a teacher was aware of or implemented a specific stage and 0 if otherwise.

**Table 1:** Variables description.

Variables	Explanation	Sign
**Dependent variables**		
Awareness	Ordinal: Number of stages a teacher was aware of	
Implementation	Ordinal: Number of stages a teacher implemented	
**Explanatory variables**		
Gender	Binary: 1 if female, 0 male	-
Qualification	Categorical: 1 Diploma degree, 2°, and 3 masters	+
Experience	Categorical: Below 5, 6–10, 11–15, 16–20 and above 20	+
Challenge	Binary: 1 if had a challenge, 0 otherwise	-

The independent variables included gender, age, academic qualifications, teaching experience, and challenges (Table 1). The variables utilized in the study were selected based on the authors' expertise, literature background, and characteristics of the sampled CRE teachers (Østby et al., 2016; Ardi and Budiarti, 2020; Musafiri et al., 2020, 2022; Azizi and Rezai, 2022). They were grounded on the capability to envisage awareness and implementation of the FSLP framework, as specified by the previous studies. Gender, qualification, experience, and challenge are the illustrative factors of the significant awareness and implementation of the FSLP (Dida et al., 2021).

Previous studies on gender dissimilarities has labelled it as a component proficient to influencing instructors' awareness and implementation of the teaching methodologies (El-Emadi et al., 2019). Instructors' academic qualifications are well thought-out a substantial interpreter of their awareness and implementation of innovative methods of teaching (Kola and Sunday, 2015). Once a teacher is familiarized to FSLP framework, it is normal to establish awareness on the FSLP framework grounded on teachers' experience and their academic qualification. So, entirely the revealed variables shaped the illustrative components of the notable awareness and implementation of the FSLP framework.

### 2.3 Research design, sample size, and sampling strategy

We employed a cross-sectional survey methodology in the study design and implementation. The design allows for data collection at a defined time with all phenomena under study (Bowden, 2011; Zangirolami-raimundo and Oliveira, 2018). The target population was all the 646 teachers in Meru County. Random sampling was employed to select schools in the sub-counties where the CRE teachers were the participants. A table of random numbers was used to generate a list of schools provided by the participants. Proportional stratified sampling assisted in obtaining the CRE teachers to participate in this study from each sub-county (Table 2). The sample size of CRE teachers who responded to the questionnaire was determined using Slovin’s formula as indicated by (Susanti et al., 2019; Insani et al., 2020; Adi et al., 2021; Lestari, 2021). We calculated the sample size following Eq. (1).

(1) n=N/(1+Ne²)

where n is the sample size, N is the total population (for this study, 646 was the total population), and e is the margin of error (0.05 margin of error). Therefore, the sample size was 247. However, this study’s response rate was 91%, with 226 teachers.

**Table 2:** Number of CRE teachers and their sample size.

Sub-County	Imenti Central	Imenti South	Imenti North	Igembe East	Igembe West	Total
CRE teachers	102	125	140	131	148	646
Sample size	39	48	54	50	56	247
Response rate	34	43	50	47	52	226

### 2.4 Ethical consideration

The study observed the ethical rules suggested by the Board of Postgraduate Studies at the University of Embu. A research approval letter was received from the National Council for Science and Technology in the Ministry of Higher Education, Science and Technology (NACOSTI) before the data collection in the field. The processes involved undergoing a research ethics review and seeking informed consent from the Ministry of Education and County Commissioner office, Meru County. Authorization from the head-teachers of the selected secondary schools was then attained through letters (written) before undertaking the research. The study obtained an informed agreement from the participating CRE teachers. The study ensured that the principle of privacy and voluntary participation was respected.

### 2.5 Data collection

Out of the 646 teachers, we collected data from the sampled 247 teachers using a questionnaire (Table 2). Thus, 247 questionnaires were administered to the CRE teachers by the investigator. The questionnaire had questions on the awareness and implementation of the FSLP framework, variables and the information on the participants' background. The participants were thereafter requested to come into agreement before their participation in the research. A questionnaire was the most economical tool based on cost-effectiveness and time management (Shahidzade et al., 2022). The researcher implemented the survey. After a successful questionnaire administration and collection process, the response rate stood at 91%. Therefore, out of 247 administered questionnaires, 226 were successfully collected by the researcher.

### 2.6 Data analysis

The data were analyzed using STATA 15.0 software. Data cleaning and coding were executed before statistical analysis. The study employed descriptive and inferential statistics. The number of practices a teacher was aware of or implemented is a count data that could be analyzed using Poisson regression. The Poisson regression assumes that all the events have the same probability of occurrence. However, the awareness and implementation of the FSLP framework do not have the same chance of happening. The propensity of awareness of the FSLP could be different from the subsequent implementation of the framework because the CRE teachers achieve the FSLP implementation upon its awareness. The CRE teachers could have gained learners' attention upon implementing the first stage of the framework and could be willing to implement a combination of all the stages to enhance performance.

Notably, awareness and implementation of the FSLP could also differ based on participants' characteristics, including; gender, experience, academic qualification, and challenges faced. However, teachers combine these stages with improving performance compared to those who implement none, single, or few. Awareness and implementation (number of stages known and implemented by the ith teacher) were ordinal variables that could be analyzed using the ordered probit model as described by (Daykin and Moffatt, 2010; Alhassan, 2020; Kanyenji et al., 2020; Musafiri et al., 2022). Before the ordered probit modeling, the data were checked for plausibility. The independent variables were subjected to correlation and multicollinearity tests, variance inflation factors (VIF), and tolerance (1/VIF).

The model allows for estimating determinants of ordinal variables (awareness and implementation) during 1, 2, 3, 4, and 5 FSLP stages. The ordered outcome could be assessed as a latent variable Y∗, where Y∗ is the unobservable measure of CRE teachers' FSLP awareness and implementation intensity (Musafiri et al., 2022) as described in Eq. (2).

(2) $$Y_{j}^{*}=X_{j}^{i}\beta +u_{j}$$

For the i^th^ CRE teacher, where normalization is that the regressors x do not include and intercept, the awareness and implementation intensity increases with Y∗. Probability of observing a j outcome is described in Eq. (3).

(3) $$\mathrm{Pr}\;\left(\text{outcome}\;i=j\right)=\mathrm{Pr}=\left(n_{j-1}\leq X_{j}^{i}\beta +u_{j}+<\alpha_{j}\right)$$

The coefficient 1, 2… j−1 were assessed jointly with the cut points 1, 2… j where j is the number of the possible outcomes. Ui is assumed to be normally distributed with a standard normal cumulative distribution function. The ordered probit model is pooled and works under the assumption that the unobserved heterogeneity is uncorrelated with the independent variables.

## 3 Results and discussion

### 3.1 Descriptive characteristics of CRE teachers in Meru County

The descriptive characteristics of variables used in modeling are presented in Table 3. The study findings on gender revealed that 143 (63%) of the participants were female, while 83 (37%) were male. The findings agreed with Munyao et al. (2017), who reported that CRE is perceived as female oriented subject by most of the boys when selecting the learning areas.

**Table 3:** Descriptive characteristics of teachers.

Variables	Description	Frequency	%
Gender	Male	83	36.7
	Female	143	63.3
Qualification	Diploma	8	3.5
	Degree	202	89.4
	Masters	16	7.1
Experience	Below 5	98	43.4
	6 to 10	76	33.6
	11 to 15	35	15.5
	16 to 20	7	3.1
	Above 21	10	4.4
Challenge	No challenge	52	23.0
	Experiencing challenge	152	77.0

On the qualification, the findings indicate that 202 (89%) CRE teachers had a degree, 16 (7%) had a master’s degree, and 8 (4%) had a diploma (Table 3). According to the Teachers Service Commission, a diploma degree from a recognized institution is the minimum requirement to teach CRE at the secondary level (Alanazi, 2019). The study findings were in harmony with (Onovughe and Mordi, 2017), who indicated that many CRE teachers in Nigerian schools had a bachelor’s degree.

Experience-wise, 98 (43.4%) CRE teachers had less than five years of experience (Table 3). Experience is an important variable, as it could influence students' performance and utilization of teaching innovations (Gore et al., 2017). Experience is a factor, which positively influences the awareness and implementation of the FSLP framework (Table 8). The findings of this study were in harmony with (Borg and Falzon, 1990), who found that experience is a determinant factor in practicing the newly invented teaching methods.

The findings on challenges faced indicated that 152 (77%) CRE teachers faced challenges during the implementation of the FSLP framework (Table 3). This was an alarm that in-service training on the framework is necessary to address the needs of CRE teachers. The findings were in harmony with (Kimosop, 2015; Assefa and Mohammed, 2022), who established the need for in-service training to equip teachers with the necessary skills to adopt updated ways of content delivery.

### 3.2 Awareness and implementation of the FSLP framework

The awareness of the FSLP framework among CRE teachers ranged between 58.41% and 99.56% (Table 4). The lowest was under guided practice, and the highest awareness was under lesson closure. The findings indicated that most teachers were aware of the FSLP framework. The findings were consistent with (West and Deutsch, 2017; Bin-hady, 2020), who found that instructors use lesson closure highly because it provides a platform for summarizing the content.

**Table 4:** Teachers' awareness and implementation of the FSLP framework.

Variables	Awareness		Implementation	
	Frequency	%	Frequency	%
Anticipatory set	224	99.14	223	98.7
New Materials	150	66.37	121	53.5
Guided practice	132	58.41	104	46.0
Independent practice	169	74.78	154	68.1
Lesson closure	225	99.56	224	99.1

Even though the guided practice provides adequate academic support and guidance during the content delivery (Morris et al., 2021), the study revealed that it had the lowest level of awareness among the participants. This can be attributed to insufficient time since all the learning objectives are achieved in this stage. The study findings agreed with (Vidal-Abarca et al., 2010), who found that guided practice is not fully realized due to different learning styles among learners, which requires a lot of time to be addressed.

The implementation of the framework ranged between 46.0% and 99.1% (Table 4). The lowest was under guided practice, and the highest was under lesson closure. The findings indicated that most of the CRE teachers implemented the FSLP framework. The results suggested that the level of awareness of any stage of the framework among CRE teachers informed their implementation in a classroom setting.

### 3.3 The number of FSLP framework awareness and implementation among teachers

The number of stages each teacher was aware of or implemented is presented in Table 5. Each teacher was aware of at least 2 of the 5 stages. The majority of the teachers (33.3%) were aware of 3 stages. The findings suggested that teachers knew numerous stages of the 5-stage lesson plan framework. Therefore, they could implement them jointly to enhance students' performance.

**Table 5:** The number of FSLP framework awareness and implementation among teachers.

Number of stages	Awareness		implementation	
	Frequency	%	Frequency	%
0	0	0	0	0
1	0	0	0	0
2	51	22.6	54	23.9
3	75	33.2	74	32.7
4	64	28.3	63	27.9
5	36	15.9	35	15.5
**Total**	**226**	**100.0**	**226**	**100.0**

The number of stages each teacher implemented is presented in Table 5. Each teacher implemented at least 2 of the 5 stages. The majority of the teachers (32.7%) implemented 3 stages of the FSLP framework. The findings suggested that CRE teachers implemented numerous stages of the FSLP framework. Consequently, implementing the 5 stages jointly among these teachers would enhance learners' performance due to improved content delivery.

### 3.4 Multicollinearity of independent variables

The variance inflation factor (VIF) ranged between 1.15 and 2.74, with a mean of 1.94 (Table 6). The tolerance ranged between 0.365 and 0.873 (Table 6). The rho values of pairwise correlation were less than 0.1 (Table 7). Since the VIF was less than 4 and rho values less than 0.2, the independent variables were not correlated, thus credible for Ordered Probit analysis (Qu, 2007).

**Table 6:** Multicollinearity of independent variables.

Variables	VIF	1/VIF
Gender	2.74	0.365
Qualification	2.71	0.369
Experience	1.17	0.855
Challenges	1.15	0.873
**Mean VIF**	1.94

**Table 7:** Correlation of independent variables.

	Gender	Qualification	Experience	Challenges	
Gender	1.000				
Qualification	0.097	1.000			
Experience	0.027	0.048	1.000		
Challenges	-0.068	0.027	0.086	1.000

**Table 8:** Determinants of the number of 5-stage lesson plan framework stages.

Variables	Awareness		Implementation	
	Coef (std)	p-value	Coef (std)	p-value
Gender	-0.317 (0.106)∗∗	0.019	-0.229 (0.149)∗∗	0.025
Qualification	-0.558 (0.309)∗∗∗	0.007	-0.176 (0.227)∗∗∗	0.003
Experience	0.108 (0.083)∗∗	0.018	0.024 (0.017)∗∗	0.019
Challenges	0.441 (0.291)∗∗∗	0.009	0.028 (0.014)∗∗	0.023
Observation = 226 LR chi^2^ = 809.65 Prob > chi^2^ = 0.0000 Log likelihood = -205.613 Pseudo R^2 =^ 0.4245			Observation = 226 LR chi^2^ = 307.85 Prob > chi^2^ = 0.0000 Log likelihood = -307.0832 Pseudo R^2 =^ 0.0573	

∗∗p < 0.05. ∗∗∗p < 0.01.

### 3.5 Determinants of awareness and implementation of the FSLP framework

The study revealed that the LR Chi^2^ 809.65, Prob > chi2 0.000 (Table 8) was significant, suggesting that the ordered probit model was credible in analyzing the number of FSLP stages known to the teacher. Similarly, the study revealed that the LR Chi^2^ 307.85, Prob > chi^2^ 0.000 (Table 8) was significant, suggesting that the ordered probit model was credible in analyzing the number of FSLP stages implemented by a teacher.

Gender was used as a control variable where during coding it was represented by 1 and 0, male and female respectively. However, referring to the ordered probit model’s results, gender of the participants negatively and significantly influenced the awareness and implementation of the FSLP framework (Table 8). The negative ordered probit model’s result is more inclined to the female than male as indicated by the coding, 0 and 1 respectively. Therefore, the findings suggested that male teachers were more likely to be aware of and implement a higher number of FSLP framework stages than their female counterparts. The findings were essential since male teachers are good at practical lessons while females are better in theory classes (El-Emadi et al., 2019). Therefore, the increased awareness and implementation of the FSLP framework could be attributed to the need for male teachers to master delivery in theory courses. The findings agreed with (Lynott and Mccandless, 2008; Nunkoo, 2012; Lee et al., 2016; Zabadi and Al-alawi, 2016), who found that males are more outcome-oriented and realistic due to high confidence, whereas females like easy and routine tasks. Contrary to the findings, Egbo et al. (2011) asserted that females were more likely to embrace new technology and strategies compared to males.

Teachers' qualifications negatively and significantly influenced the awareness and implementation of the FSLP framework (Table 8). The negative prediction of academic qualification was unexpected. Generally, qualification increases knowledge of educational technologies, thus increasing awareness and implementation of the technological strategies (Manning et al., 2017). Therefore, the negative prediction of academic qualification could be ascribed to varied objectives, such as learners' performance, teachers' promotion, and syllabus completion opposed to awareness and implementation of the FSLP framework. Contrary to the findings, Musau and Abere (2015) found that teacher qualification does not significantly influence their teaching strategies and academic performance in the subjects.

Work experience positively and significantly (β = 0.108, p = 0.018) influenced teachers' awareness and implementation (β = 0.024, p = 0.019) of the FSLP framework (Table 8). The finding suggests an increase in teachers' experience will lead to a corresponding increase in teachers' awareness and implementation of FSLP. This finding can be attributed to the long exposure of teachers to the model, hence increased awareness and implementation. This result is consistent with (Getachew et al., 2016; Podolsky et al., 2019), who found that teacher's experience was an influential positive factor in the teacher's awareness and implementation of any new teaching strategy.

Challenges positively and significantly influenced the awareness (β = 0.441, p = 0.009) and implementation (β = 0.028, p = 0.023) of the FSLP framework (Table 8). The finding suggests that exposure to challenges increases teachers' awareness and implementation of the FSLP framework (Table 8). The study showed that only 16% of CRE teachers were aware of and implemented this framework’s stages (Table 5). This implied that these teachers faced challenges in fully embracing the FSLP framework. In agreement with the findings, s found that diverse challenges faced by teachers while preparing and initiating their instructional practices were an influential positive factor in teachers' awareness and implementation of newly invented content delivery methods.

## 4 Conclusion and policy recommendations

The FSLP framework significantly affects the teaching of CRE in Eastern Kenya. We assessed the number of FSLP stages a teacher was aware of or implemented and the determinants of awareness and implementation of the FSLP framework. The awareness level and implementation of the FSLP framework were high. Our findings revealed that the gender of the respondent and academic qualification negatively while working experience and challenges faced positively determined the FSLP framework awareness and implementation among CRE teachers.

Based on our findings, we highlight three policy recommendations. First, the study recommends a framework policy targeting the awareness and implementation of the FSLP framework to strengthen teachers' knowledge of the teaching strategy. This policy is achievable through enhanced in-service training, organized seminars, and workshops on jointly implementing the five stages of the FSLP framework. Therefore, there is an imperative need to increase in-service education activities to equip teachers with the latest skills to grow professionally.

Second, the study suggests that the mentioned framework policy should be specific, clear and tailored to perceptions, ideas and opinions of the CRE stakeholders to enhance awareness and implementation of new teaching strategies such as the FSLP framework. Additionally, the clarity of the framework’s policy formulates vibrant goals and expectations, which enhance scaffolding for learners' academic growth and institute reliance between learners and the teacher. When teachers make teaching and learning expectations and objectives clear to their learners, they improve learners' capabilities to meet those expectations.

Third, the study recommends a quality assurance mechanism to measure the curriculum implementation by the CRE teachers. The main purpose of this mechanism is to ensure that CRE teachers meet a high truthfulness in implementing the CRE curriculum. Hence, CRE teachers' strengths and weaknesses are addressed by the responsible departments in the Ministry of Education. Initiating the above recommendations could improve FSLP framework awareness and implementation among CRE teachers.

The study has specific limitations; for instance, the succeeding research should question the relationship between gender and awareness of the FSLP and purposely try to comprehend how the two variables influence the application of the FSLP framework. Additionally, the study suggests further investigation to analyze female and male CRE teachers' awareness of the FSLP framework through a suitable model such as bivariate analysis. Furthermore, other investigation methods, such as interviews, can recognize what CRE teachers contemplate regarding the FLSP framework.

## Declarations

### Author contribution statement

Saoke V.O.; Musafiri C.M.: Conceived and designed the experiments; Performed the experiments; Analyzed and interpreted the data; Wrote the paper.

Ndwiga Z.N.; Githaiga P.W: Conceived and designed the experiments; Performed the experiments; Contributed reagents, materials, analysis tools or data; Wrote the paper.

### Funding statement

This research did not receive any specific grant from funding agencies in the public, commercial, or not-for-profit sectors.

### Data availability statement

Data will be made available on request.

### Competing interest statement

The authors declare no conflict of interest.

### Additional information

No additional information is available for this paper.
