# Supplementary material for: Determinants of awareness and implementation of five-stage lesson plan framework among Christian Religious Education teachers in Meru County, Kenya
Source: Heliyon. 2022 Oct 26;8(11):e11177. doi: 10.1016/j.heliyon.2022.e11177 (PMC9634273; doi:10.1016/j.heliyon.2022.e11177)
Supplement: Supplementary file [file mmc1.docx]

**Research questionnaire**

I am implementing a study project entitled ***Assessment of the five-stage lesson plan framework application in teaching Christian Religious Education in Meru County****.* You are among several teachers in the County selected for this study. This study seeks to investigate the awareness and implementation of the Five-Stage Lesson Plan framework among Christian Religious education teachers in the County. The information provided will be treated with the utmost confidentiality. Your assistance in answering the questions truthfully and accurately will be highly appreciated. Participation in the study is voluntary, and the information collected will be reported as a statistical summary without disclosing the teacher’s details.

**Section A: Questionnaire identification**

1. The questionnaire number…………
2. County……………
3. Sub-County…………
4. Ward………….
5. School……….
6. Type of school 1 public, 0 private

**Section B: Teacher profile**

1. What is your name (optional)……………………………………?
2. What is your gender? 1 female, 0 male
3. What is your age bracket? 1if below 35, 2 if 35-50, and 3 if above 50
4. What is your level of education? *1 if Diploma, 2 if Degree, and 3 if master*
5. For how long have you been teaching Religious Christian education? *1= Below 5, 2= 5-10, 3=11-15, 4= 16-20, 5= 21 and above*

***Section C Lesson plan***

1. What is a lesson plan? .................................................................................................................................................
2. What is the primary significance of a lesson plan in teaching? ...............................................................
3. Do you always prepare a lesson plan? 1 if yes and 0 if no

**Section D: Five-stage lesson plan framework**

1. Are you aware of the five-stage lesson plan framework? 1 if yes, 0 if no
2. How many stages are involved in the lesson plan?
3. What is a five-stage lesson plan framework?
4. Name the five lesson plan stages?
5. Among the five stages listed below, kindly select the one you are aware of.

*Tick multiple is only the stages you are aware of*

| ***S No*** | ***Stage*** | ***Aware of the practice*** |
| --- | --- | --- |
| *1* | Anticipatory set |  |
| *2* | Introduction of new materials |  |
| *3* | Guided practice |  |
| *4* | Independent practice |  |
| *5* | Lesson closure |  |

1. *Do you implement any of the five-stage lesson plan stages in class? 1 if yes and 0 if no*
2. Among the five stages listed below, kindly select the one you are implementing in class*?*

*Tick multiple that is only the stages you implement in class*

| ***S No*** | ***Stage*** | ***Implement the stage in class*** |
| --- | --- | --- |
| 1 | Anticipatory set |  |
| 2 | Introduction of new materials |  |
| 3 | Guided practice |  |
| 4 | Independent practice |  |
| 5 | Lesson closure |  |

**Section E: Lesson plan implementation challenges**

1. Do you experience challenges during the implementation of the five-stage lesson plan? 1 if yes, 0 if no
2. If yes to the section E i above, list the main challenges experienced in the implementation of the five-stage lesson plan ……………………………………………………………………………………………………………………………………………………………………………………………………………………………………………………………

**Comments**

Provide any additional information or comments regarding the study

……………………………………………………………………………………………………………………………………………………………………………………………………………………………………..

**Thank you**
